# Local versus national banking development in Europe: who is the winner?

**DOI:** 10.1007/s40821-022-00233-0

**Published:** 2023-01-28

**Authors:** Francesco Fasano, Maurizio La Rocca

**Affiliations:** 1 Department of Business Administration and Law, University of Calabria, Campus of Arcavacata, 87036 Rende, CS Italy; 2 Department of Business Administration and Law, University of Calabria, Campus of Arcavacata, 87036 Rende, CS Italy

**Keywords:** Capital structure, Debt, SMEs, Banking development, G30, G32

## Abstract

This article investigates the role of local banking development, per se, in European SME debt policies, using a cross-country dataset. Moreover, it assesses whether the debt policies of SMEs are mainly driven by local or national banking development. We find that higher levels of local banking development increase the amount of debt used by SMEs. Results suggest that national banking institutions reduce the influence of local banking sectors on SME financial behavior. Several robustness and further tests validate our results, suggesting that whilst the process of international integration and digitalization of financial markets, local banking development is still relevant despite the moderating role of national banking policies. Consequently, policy makers should first and foremost allow headquarters and central banks to better support local banks in integrating national banking policies in order to reduce the financial constraints on SMEs and spur their economic growth.

## Introduction

The influence of financial institutions on firm policies is one of the most important topics in corporate finance. Noteworthy authors found that the development of the financial system^1^ at the national level has a crucial impact on corporate growth processes (Allen & Gale, 2000; Demirgüc-Kunt & Maksimovic, 1998; Levine, 2002; Rajan & Zingales, 2001). These authoritative papers generated much academic interest in this field, attracting the attention of researchers all over the world. This resulted in a rapid increase of papers that investigated how financial institutions influence firm business in several respects. One of these aspects concerns the role of local financial development on corporate growth (Guiso et al., 2004; Kendall, 2012; Schmidt, 2021). The pioneer paper in this field is the worthwhile work of Guiso et al. (2004), which has been enormously influential and of inspiration for subsequent directions of research. Thanks to this contribution, it turned out that despite the contemporary integration and digitalization of the financial markets (Orlowski, 2020; Fasano & Cappa, 2022), local banking development (LBD)^2^ significantly matters for firm growth. Moreover, the authors interestingly observed that the development of local banking sectors matters only for Small and Medium-sized Enterprises (SMEs), which face high asymmetric information problems in debt contract negotiations (Berger & Udell, 1998). Indeed, SMEs’ information opacity makes such firms strongly rely on the development of the local banking system, which can alleviate their financial constraint problems (Alessandrini et al., 2009a; Beck et al., 2005; Pollard, 2003). This is because the proximity between the SME and the outside lenders mitigates asymmetric information difficulties, as evidenced by Petersen and Rajan (2002). Face to face relationships allow banks to acquire soft information^3^ that, differently from hard information, is difficult to standardize and is crucial in lending decisions (Boot, 2000). This is remarkable, since the access to finance is one of the most pressing problems following the financial crisis, as evidenced by the European Central Bank,^4^ and is particularly crucial in the recent context of the coronavirus (Covid-19) crisis (Fasano et al., 2022). Therefore, LBD might reduce the obstacles to the funding of SMEs, which have a key role in economic growth as they represent 99% of businesses in the European Union.^5^ Given the importance of SMEs, starting with the aforementioned contributions that have gone down into the financial literature history, some articles studied how well-functioning financial sectors influence the use of debt (González & González, 2008; La Rocca et al., 2010; Palacín-Sánchez & Di Pietro, 2016; Utrero-González, 2007).

In this paper, we provide a contribution to this body of literature, studying the effect of LBD on the debt decisions of SMEs, using a unique large dataset composed of SMEs from six different European countries.^6^ Differing to previous studies, which investigated the effects of LBD in a single country setting of analysis, we carry out a cross-country analysis that has the advantage to control for different institutional environments in a wide European context, allowing for better generalizability of the results.

In addition, our analysis provides another important originality in studying whether SMEs rely more on LBD or national banking development (NBD). NBD refers to the banking development at the country level (Gardener et al., 2011). The World Bank Development Indicator^7^ quantifies NBD in terms of domestic credit provided by the financial sector as a share of GDP, referring to the measures of banking sector depth and financial sector development.^8^

In particular, we investigate the moderating role of NBD on the relationship between LBD and debt policies. Despite the extant literature suggesting that both local and national banking systems are important, no study has yet investigated which of the two better drives SME debt policies. This interesting aspect is relevant as the different influence of one or the other level of banking system has crucial implications for both SMEs and policy makers, particularly in the actual highly integrated international financial sectors.

The results show that LBD significantly and positively affects the debt decisions of European small businesses. SMEs set in local environments with more developed banking institutions use more external debt. Moreover, the findings also suggest that the development of national banking sectors conditions the effect of local ones on SME debt decisions. It seems that when the national banking setting is more developed, the relevance of local banking institutions is lower. Vice versa, when the national banking sector is less developed, the importance of local banking sectors is greater. The implications of this research are crucial. First, similarly to the findings of previous single country studies, it confirms at the European level that, despite the international integration of financial markets, LBD still matters for SMEs. Consequently, government should carefully consider the development of both local and national banking sectors in order to spur SME growth. Second, the findings give an extraordinary importance to the actions of European governments, which should primarily improve national channels of funding in order to mitigate SME financial constraint problems. Several further and robustness tests were performed to validate the results.

The remainder of the paper is structured as follows. Section 2 describes the main literature and the research hypotheses. Section 3 explains data, methodology, and variables, while Sect. 4 shows the descriptive statistics and correlations. Section 5 illustrates results, further tests and robustness tests. The paper ends with conclusions in Sect. 6.

## Theory and hypotheses development

### Local banking development and SMEs’ debt policies

There is broad consensus in the extant literature that debt is a fundamental dimension that plays a significant role in shaping firm policies. The importance of debt has been previously studied beginning with noteworthy contributions (see the review of Harris & Raviv, 1991). It has been sufficiently documented that debt financing is more difficult to obtain when asymmetric information between borrowers and lenders is relevant, and lending costs generally increase too, especially for SMEs (Berger & Udell, 1998). In this context, the development of the banking system is a strong tool that could mitigate SME financial restrictions (Beck et al., 2005; Demirgüc-Kunt & Maksimovic, 1998; Pollard, 2003) and prevent bankruptcy (Arcuri & Levratto, 2020). These articles suggest that SMEs seeking external funding are particularly influenced by the quality of the banking system in which they are embedded. In more detail, SMEs in countries with well-developed banking sectors are more likely to obtain external financing than SMEs in countries with lower levels of banking development.

The above-reviewed contributions interestingly highlight the relevance of credit institutions for corporate debt policies. However, they investigate the role of banking development at the country level, ignoring that at the local level there are also different degrees of banking development (Guiso et al., 2004) that could affect SME debt decisions (Pollard, 2003). Inspired by such arguments, a novel and attractive line of study has started investigating how LBD affects firms’ financial behavior. In this stream of research, Alessandrini et al. (2009a), based on the Italian context, reveal that the physical distance between the firm and its financiers obstructs credit provision, especially for small firms. A year later, La Rocca et al. (2010) carried out an empirical analysis using the same Italian context. In this paper, the authors point out that higher levels of provincial banking development increase the use of debt by SMEs. Similarly, some authors in Spain observe that the differences in SMEs debt levels lie in the differences in the local banking institutions (González & González, 2008; Palacín-Sánchez & Di Pietro, 2016; Utrero-González, 2007). Thus, in general more developed local banking sectors facilitate the acquisition of soft information on small entrepreneurs (Agarwal & Hauswald, 2010; Howorth & Moro, 2006), reducing information asymmetries between the bank and the SME. However, despite the evidences of the latter studies, Siranova and Rafaj (2021), recently suggest that the’too-much-branching’ phenomenon increases inefficiency of individual banks and reduces the cost of capital, consequently discouraging loan requests. In a similar vein, Hernández-Cánovas and Martínez-Solano (2010) highlight that bank relationships reduce the financial flexibility for the borrowers and are associated to higher lending costs.

Our work aims to provide further investigation in this field of research by focusing on a wide European context of analysis. Based on the main idea that soft information about SMEs is less available to banks in a context of centralized bank industry, where the distance from local firms is higher (Stein, 2002), we expect that SMEs that have easier access to external funds report higher levels of indebtedness. Therefore, we hypothesize that also in a cross-country context the proximity between the SME and the banking sector could increase the access to debt financing (*Hypothesis 1*):

#### Hypothesis 1

Local banking development has a positive effect on European SME use of debt.

### The moderating effect of national banking development on local banking development

The above arguments that led to the development of the first research hypothesis highlight how firms debt decisions are determined by factors that are related to local banking sectors. Antecedents in literature suggest that institutional factors at the national level also have a key role on corporate debt policies (Agarwal & Mohtadi, 2004; Fan et al., 2012). Noteworthy contributions focus on this field of research, which is a core topic in the financial studies (Fan et al., 2012). Mayer (1990) and Rajan and Zingales (1998, 2001) find that higher levels of financial development reduce the cost of external finance. Chittenden et al. (1996), focusing on SMEs, point out that the access to capital markets is an important determinant of debt choices. Likewise, Fan et al. (2012), similarly to Demirgüc-Kunt and Maksimovic (1998), argue that country factors, as financial markets, have a key role in capital structure determinants, affecting the availability of debt. Moreover, Giannetti (2003) suggests that debt ratios are influenced by the degree of the financial sector development. All these contributions investigate the role of the banking systems on corporate debt policies focusing at a national scale and suggesting that the efficiency of the national banking system mitigates the problem of asymmetric information in similar vein as described in the literature reviewed in the preceding sub-Sect. 2.1 on a local basis. It is thus noticeable that two streams of research present areas of overlapping and interconnections: one studying the role of local banking sectors, the other studying the role of national banking sectors on corporate debt policies. Nevertheless, although access to capital markets is a major concern for SMEs in any country, previous contributions do not shed light on a still unresolved critical issue: is there a relationship between the effects of LBD and NBD on the debt choices of SMEs? In other words, does the banking institutional context of a country moderates the role of LBD on corporate debt decisions? In the face of the discussion in the financial research, the questions naturally arise. From one side, one could expect that the totality of the provincial banking developments builds up the degree of NBD. In other words, NBD would be generated from the sum of each single provincial level of banking development (bottom-up effect), and thus, NBD would not affect the role of local banking sectors. Local banks are independent financial service providers with discretion power on credit access. Thus, the degree of financial development at the national level would be driven by local sectors. However, from another perspective, the whole NBD could drive the development of the underlying local banking institutions (top-down effect). Thus, national banking institutions (i.e. National Central Banks) would shape the impact of local banking sectors on firms’ debt decisions. Specifically, bank headquarters can be at the core of main guidance, supervision and control on local banking sectors in such a way that reduces the local managerial discretion. Thus, the central leadership provides the main guideline and directions to be followed by local units. It is well known that loan decisions’ criteria and resource allocation are run by banks’ centralized headquarters (Alessandrini et al., 2009b). Banks’ headquarters influence the operational activities of the financial intermediation of local branches (Ozyildirim & Onder, 2007). The headquarters provide rules, monitoring, advice and constraints on the managerial discretion of local branches in providing credit in the local market. They typically operate nationwide and their supervision and regulatory activities are likely to affect the debt contracting at the local level.

At the same time, national central banks shape countries’ banking sector decisions at both national and local level (Wellink et al., 2002). In some cases, as interestingly highlighted by Guiso et al. (2004), the central bank “used to authorize the opening of new branches”, playing a key role in LBD. Recent compulsory measures implemented by central banks to regulate local branches’ activities during the recent Covid-19 emergency constitute a clear example (Cavallino & De Fiore, 2020). Operations of local branches reflect both headquarters’ rules and central banks’ national guidelines that, in turn, reflect the political realities of a country (Posen, 1995). Therefore, companies financing is influenced by LBD that is in turn shaped by the correspondent NBD at the country-level.

Moreover, despite the presence of significant differences across provinces (Guiso et al., 2004; Utrero-González, 2007), the LBD of European provinces is still likely to be a by-product of the national banking system. Provinces represent a sub-national level under the same institutional conditions (e.g., regulation, legislation, tax, etc.) and below the policies of national headquarters and central banks. Therefore, we suppose that the top-down effect prevails as national banking institutions drive loan policies throughout the national territory. Consequently, we hypothesize that the NBD moderates the effect of LBD on the corporate debt strategies of SMEs, and not vice versa. Thus, on the basis of this reasoning, we expect that (*Hypothesis 2*):

#### Hypothesis 2

National banking development moderates the effect of local banking development on European SME use of debt.

## Research design: data, methodology, and variables

### Sample and context

The study is based on a sample of non-financial SMEs from six European countries: Finland, France, Germany, Italy, Spain and the United Kingdom.^9^ SMEs are selected according to the European Commission’s definition^10^ in terms of employees (fewer than 250), annual turnover (lower than EUR 50 million) or annual balance sheet total (not exceeding EUR 43 million). The dataset is derived from several sources. Firm-specific data are collected from the Amadeus database of Orbis from Bureau Van Dijk, which is the most extensive database of private and public companies across Europe, as it contains detailed and well-harmonized accounting, financial and business information for European SMEs. Finally, data regarding the enforcement, domestic product (GDP) and population at the provincial level come from the national statistical institutes of the single European countries.^11^ We imposed restrictions on the data as follows. First, we selected only firms with accounting information available. Then, we limited the impact of the outliers, winsorizing all the control variables at the first and 99th percentiles. Lastly, observations with errors (e.g., non-positive values for total book assets) and zero sales were removed. Our final database makes an unbalanced panel set of 285,974^12^ firm-year observations over the period 2004–2010.

Our paper analyzes bank-based economies as well as one market-based economy (i.e., the UK). Despite the presence of the European banking union created in 1993 to guarantee the stability of the banking sector in the euro area, each single European country has its own characteristics in terms of banking system. In France, the deregulation process in 1984 had a key role on building the actual banking organization, which is composed on commercial banks that are spread throughout the territory and some of them are among the largest banks in the world (Lepetit et al., 2016). In Germany it is relevant the presence of regional savings banks and cooperative banks that are particularly important for firms compared to centralized banks, while in UK there is a centralized system, with important banking centers as the one in London (Flögel & Gärtner, 2018). In Spain, the presence of large commercial banks coexists with saving banks which expanded their branchnetwork after the 1988 reform (Gärtner & Fernandez, 2018). Finland has historically had a large number of bank branches related to its population (Zardkoohi & Kolari, 1994), while it is now experiencing a period of high development of fintech that could change the role of branches (Hundal & Zinakova, 2021). Finally, in Italy national banks dominate the market, but local banks also have an important role in the banking system (Fasano & Deloof, 2021). All these arguments shed light on the fact that the local banking sectors differ from country to country, but also national banking policies vary, with central banks and banking headquarters that have to deal with the characteristics of the nation to which they belong, suggesting the relevance of studying the impact of LBD for firms and the moderating role of NBD.

### Variables

This study uses as dependent variable *Debt* that is a proxy for the amount of bank debt used by SMEs. Following the capital structure literature (e.g., Rajan & Zingales, 1995), the financial level of indebtedness is calculated by the ratio of long-term and short-term interest-bearing bank debt scaled by total assets. As the first independent variable, following the approach of Guiso et al. (2004), Benfratello et al. (2008), Alessandrini et al. (2009a), La Rocca et al. (2010) and others, we measure *LBD* considering the number of national bank branches scaled to 1,000 inhabitants in the province, a well-known proxy for LBD. This variable is widely used in the previous studies as it clearly explains the dimension of the banking development at the local level. Data concerning LBD are collected from the banks of the individual European countries.^13^The banks provide information about the density of bank branches per province, corresponding to the NUTS3 areas in the EU classification according to the statistical office of the European Union (Eurostat dataset). We use bank branch density as our measure of LBD because, bank debt still remains the most used source to finance fixed assets investments in Europe.^14^*NBD* is calculated, according to the World Bank approach, as the total domestic credit provided by the financial sector to the private sector by banks as a percentage of GDP. Previous empirical literature has used this measure as a standard proxy of banking development at the national level (e.g., Clarke et al., 2006; Nikoloski, 2013) and is suggested as a measure of the extent with which private sector agents easily get external credit. In order to avoid multicollinearity problems, the variable *NBD* is not measured as an aggregation of local level and is thus not calculated considering the national density of bank branches. The database of “World Development Indicators” provided by the World Bank makes our measure of NBD available. Table 1 synthetizes the variables description.

**Table 1:** Variables description

Dependent variable	Calculation
Debt	(Long-Term Bank Debt + Short-Term Bank Debt)/Total Assets
Explanatory variables	
LBD (Local Banking Development)	(Total Bank Branches at provincial level × 1000)/population at provincial level
NBD (National Banking Development)	Domestic credit provided by financial sector by banks (% of GDP)
Interests	Financial interests/Debt
Cash holdings	Cash & cash equivalents/total assets
Trade credit	(Receivables – payables)/total assets
ROA	EBIT/Total Assets
Size	ln(total assets)
Tangibility	Tangible Assets/Total Assets
Age	Year–year of birth
Different tax shield	(EBITDA—EBIT)/total assets
Firm growth	(Sales t – Sales t -1)/Sales t -1
GDP growth	[(real GDP at provincial level)_*t*_ – (real GDP at provincial level)_*t -1*_]/(real GDP at provincial level)_*t -1*_
Enforcement	Mean time required to enforce a right at the provincial level

We also include a number of firm-specific variables that may influence/interact with the effects we want to analyze. *Cash Holdings*, which is the ratio between cash and cash equivalents scaled by total assets (e.g., Almeida et al., 2004; Ozkan & Ozkan, 2004). *Trade Credit* is calculated as receivables minus payables over total assets (e.g., Deloof & La Rocca, 2015). This variable shows the net investment of SME in trade credit. *ROA* is a variable broadly used in the financial literature to measure SME performance. It is calculated as earnings before interest and taxes (EBIT) scaled by total assets. We use an accounting-based measure as no information about the market value of small businesses is available. Firms that are more profitable are likely to have a proactive approach versus financial strategies. *Size* is measured as the logarithm of total assets. Larger firms typically have a bargaining power with their lenders or suppliers. *Tangibility* is the ratio of tangible fixed assets to total assets. Tangible assets may increase firms’ financial capacity as they are used as collateral. *Age* is calculated as year minus year of incorporation. Older firms have a long history that reduces information asymmetries and facilitates credit provision. *Different Tax Shield* is calculated as earnings before interest, taxes, depreciation and amortization minus earnings before interest and taxes (EBITDA—EBIT) scaled by total assets. This variable is particularly important to control for the different tax regimes of the European countries. *Firm Growth* is measured as sales in year (t) minus sales in year (t−1). Growing SMEs generally require more financial resources (Binks & Ennew, 1997). We also control for provincial characteristics that may affect the results. *GDP Growth* is measured as the growth in real GDP at the provincial level from year (t−1) to year (t). *Enforcement* represents the time required to enforce a right, in terms of number of days necessary to conclude a court case, and takes into account the efficiency of the law courts at the local level (Agostino et al., 2012). Industry and year fixed effects using dummies are also included in the econometric model.

### Methodology

Based on an unbalanced panel sample, we investigate our hypotheses using the panel fixed effects (FE) technique, whose advantages have been clearly explained by the extant literature (Bliese et al., 2020; Gormley & Matsa, 2014). Before launching our regressions, we first ran the Hausman test, which suggests that the FE model better fits our data. Additionally, we ran a parm test which suggests that time FE are needed. The empirical model investigated is the following:

While for the second hypothesis, we added the variable measuring NBD and its interaction term with the variable measuring LBD, obtaining the following model:

$$Debt = f\left( {LBD,NBD,LBD \times NBD,{\text{ control variables}}} \right)$$

We provided several robustness to verify that the results are not driven by econometric technique. Following the approach of Deloof and La Rocca (2015), as a robustness test we use the ordinary least squares based on clustered standard errors (OLS cluster). As additional robustness exams, we perform the two stage least-squares (2SLS) regressions for a sub-sample of Italian SMEs using the same instrumental variables as in Guiso et al. (2004). Then, we applied a placebo test to make sure that the high number of observations does not lead to false statistically significant results. Finally, we run other robustness tests, also using a different proxy of LBD.

## Descriptive statistics and correlations

Table 2 shows the descriptive statistics in terms of mean, standard deviation, minimum value, 25th, 50th (median), 75th percentiles and maximum value for all the variables.

**Table 2:** - Descriptive Statistics for the sample

	Mean	Sd	Min	p25	Median	p75	Max
Debt	0.202	0.197	0.000	0.021	0.150	0.339	0.847
LBD	0.736	0.260	0.172	0.568	0.708	0.880	2.253
NBD	1.065	0.376	0.637	0.815	0.885	1.360	1.921
Cash holdings	0.097	0.131	0.000	0.009	0.043	0.133	0.991
Trade credit	0.127	0.206	-2.689	0.002	0.107	0.233	0.999
Interests	0.129	0.215	0.000	0.018	0.061	0.157	0.931
ROA	0.060	0.101	-0.317	0.017	0.048	0.098	0.434
Size	8.697	0.903	1.946	8.034	8.706	9.367	10.669
Tangibility	0.226	0.198	0.000	0.067	0.172	0.335	0.996
Age	2.917	0.762	0.000	2.485	3.045	3.434	5.347
Different tax shield	0.039	0.027	0.002	0.018	0.033	0.054	0.104
Firm growth	-0.056	0.319	-17.054	-0.146	-0.032	0.077	0.947
GDP growth	0.240	0.019	0.136	0.233	0.240	0.255	0.271
Enforcement	0.082	0.041	0.023	0.040	0.052	0.121	0.139

Descriptive statistics show that our dependent variable play a very important role in the financing of European SMEs. In particular, on average debt represents 20.2% of total assets. Moreover, the standard deviation of *Debt* (0.197) indicates a large variability of the dependent variables across the SMEs in the sample. Table 2 shows that there is also substantial variation with respect to both LBD and NBD, while the values for the control variables are in line with the existing financial literature studies. We also report descriptive statistics by country and sector (Table 12 and 13 in the appendix, respectively), observing that the use of debt is relevant for all the European countries (to a lesser extent only for France) and all industries.

Additionally, we report descriptive statistics of the variable *Debt* for high and low levels of both *LBD* and *NBD* based on the median.^15^ The results, which are shown in Table 8 and 9 in the appendix, interestingly reveal that higher levels of both *LBD* and *NBD* increase the use of debt. This demonstrates that different degrees of our key independent variables influence the debt policies of SMEs. Moreover, we sort the whole sample into four sub-samples according to low and high levels of *LBD* and *NBD* both based on median values.

Furthermore, we show (in Fig. 2 in the appendix) the trend of the *Debt* during the years, observing a mostly stable trend throughout the period examined and suggesting that the influence of the economic cycle should not be very decisive.

Table 3 reports the correlation matrix of the variables.

**Table 3:** Correlation matrix

		(1)	(2)	(3)	(4)	(5)	(6)	(7)	(8)	(9)	(10)	(11)	(12)	(13)	(14)	VIF
(1)	Debt	1.00														
(2)	LBD	0.06	1.00													1.75
(3)	NBD	0.06	0.62	1.00												2.65
(4)	Cash hold	− 0.33	0.03	0.07	1.00											1.22
(5)	Trade credit	− 0.03	0.21	0.26	− 0.06	1.00										1.21
(6)	Interests	0.42	− 0.01	− 0.01	− 0.20	− 0.08	1.00									1.34
(7)	ROA	− 0.23	− 0.00	− 0.01	0.27	0.09	− 0.12	1.00								1.14
(8)	Size	0.20	− 0.01	− 0.04	− 0.18	− 0.15	0.48	− 0.10	1.00							1.17
(9)	Tangibility	0.24	0.01	0.05	− 0.21	− 0.22	0.13	− 0.11	0.12	1.00						1.44
(10)	Age	− 0.05	− 0.03	− 0.04	0.00	− 0.03	0.02	− 0.04	0.16	0.07	1.00					1.06
(11)	Dif. Tax Sh	0.05	− 0.03	− 0.03	− 0.08	− 0.12	− 0.01	− 0.07	− 0.11	0.44	− 0.04	1.00				1.31
(12)	Firm grow	0.01	0.01	0.03	− 0.01	0.00	− 0.02	− 0.17	− 0.07	0.05	0.12	0.03	1.00			1.06
(13)	GDP grow	− 0.12	0.19	0.18	0.08	0.09	− 0.06	− 0.02	− 0.06	− 0.17	0.03	− 0.10	0.01	1.00		1.09
(14)	Enforcement	0.12	− 0.22	-0.58	− 0.19	− 0.12	0.09	− 0.08	0.19	− 0.01	0.02	− 0.06	0.00^+^	− 0.05	1.00	1.73

Notes: Industry and year dummies are not reported. All the correlations different from 0.00 are statistically significant at the 0.01 level

All the correlations different from 0.00 are statistically significant at the 0.01 level, which could pose a multicollinearity problem. We calculate the variance inflation factors that estimate how much the variance in the regression coefficients is inflated due to multicollinearity. The maximum VIF in the model is 3.86 (mean of 1.40) that is far below the generally accepted cut-off of 10 (or, more prudently, 5) for regression models (D'Angelo et al., 2022; Pinelli et al., 2020), ensuring the validity of the variables significance.

## Empirical results

### Local banking development and SMEs’ debt strategies

Table 4 shows the results of the study using the panel FE method. Column 1 reports the effect of LBD on SMEs debt, column 2 adds the variable NBD, while column 3 includes the interaction term that is our moderating variable based on the variable *LBD* multiplied by the variable *NBD.* An F-test supports the hypothesis regarding the joint significance of *LBD* and its interaction term. The p-values are based on heteroscedastic robust standard errors.

**Table 4:** Main model: results concerning the effect of LBD on SME debt policies and the moderating effect of national on LBD

Estimation technique:	(1) Panel FE	(2) Panel FE	(3) Panel FE
Dependent Variable	Debt	Debt	Debt
LBD	0.020***	0.013***	0.036***
	(0.004)	(0.004)	(0.008)
NBD		0.020***	0.039***
		(0.003)	(0.007)
LBD × NBD			-0.018***
(interaction)			(0.006)
Cash holdings	− 0.076***	− 0.074***	− 0.074***
	(0.004)	(0.004)	(0.004)
Trade credit	0.065***	0.065***	0.065***
	(0.003)	(0.003)	(0.003)
Interests	0.109***	0.109***	0.109***
	(0.009)	(0.009)	(0.009)
ROA	− 0.229***	− 0.226***	− 0.226***
	(0.005)	(0.005)	(0.005)
Size	0.074***	0.073***	0.072***
	(0.002)	(0.002)	(0.002)
Tangibility	0.063***	0.063***	0.062***
	(0.005)	(0.005)	(0.005)
Age	− 0.005**	− 0.013***	− 0.014***
	(0.002)	(0.003)	(0.003)
Diff. tax shield	0.123***	0.132***	0.135***
	(0.025)	(0.025)	(0.025)
Firm growth	0.012***	0.012***	0.012***
	(0.001)	(0.001)	(0.001)
GDP growth	0.715***	0.510***	0.476***
	(0.105)	(0.105)	(0.105)
Enforcement	0.056	0.094*	0.139***
	(0.050)	(0.050)	(0.051)
Adj. R2	0.097	0.097	0.097
Observations	285.974	285.974	285.974

Industry and year fixed effects are the controls. The superscripts denote significance as follows: *p < 0.10, **p < 0.05, ***p < 0.01. Regressions report heteroscedastic robust standard errors in brackets

Our main results reveal that LBD significantly affects our variable *Debt*. The results support the argument that the close proximity between SMEs and provincial bank branches stimulates credit provision in the European countries. Therefore, the banking development of the geographical area where a SME resides increases the availability of financial resources, since bank branches can easily obtain deep information and reduce information asymmetries. Additionally, the negative coefficient of the variable *Cash Holdings* confirm that European SMEs use financial borrowing as a substitute for cash (Fasano & Deloof, 2021). The findings corroborate those of La Rocca et al. (2010) and Alessandrini et al. (2009a). However, differing from previous contributions, we find for the first time that this positive important effect exists not only at a single country, but at the European level as well. To sum up, we find that LBD matters in the European countries even in a globalized world, allowing for a better generalization of the noteworthy findings of Guiso et al. (2004) and other single country studies above mentioned.

### The moderating effect of national banking development on local banking development

Turning to the second question of the paper, this paragraph investigates through a moderation analysis whether the debt policies of SMEs are more influenced by LBD or NBD.

**Fig. 1:**
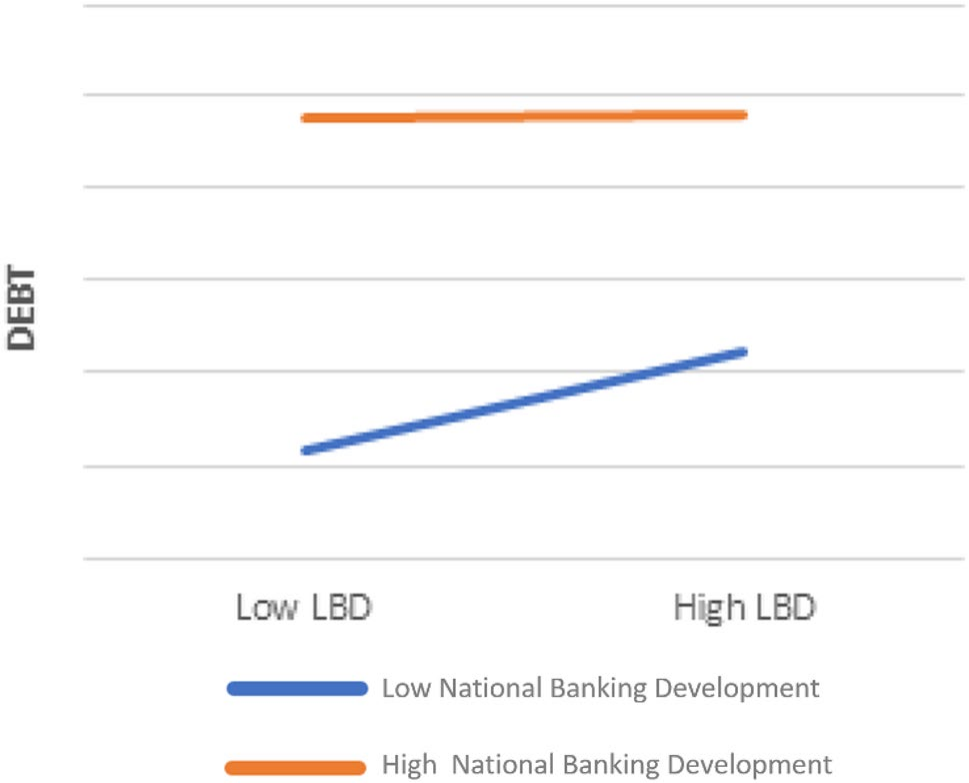
Marginal effect of LBD on debt conditioned by NBD

**Fig. 2:**
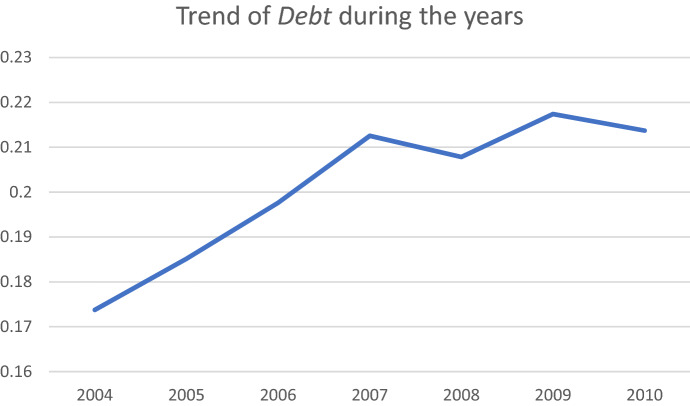
Trend of Debt during the years

Table 4 estimates in column 3 the marginal impact of the variable *LBD* for different levels of the variable *NBD*, in order to scrutinize whether the effect of local banking sectors differs in magnitude according to different levels of national development. To calculate the interaction effects between two continuous variables it is indispensable to use a graph, since the regression coefficients do not provide a correct interpretation of the marginal effect studied. A graph clearly shows the partial effect of local banking sectors on SME debt policies conditional for high or low levels of *NBD*. Therefore, for a better understanding of the results, the marginal effect of LBD on debt conditioned by NBD, resulting from regressions of Table 4, is graphically shown in the following Fig. 1.^16^

Table 4 and Fig. 1 show that the role of local banking institutions on SME debt decisions differs in magnitude according to the different levels of NBD. In particular, the interaction term, which we measure at the 95% confidence interval in regressions, is negative and statistically significant, indicating that the positive effect of LBD on SME debt tends to decrease as the level of NBD rises. Therefore, national banking sectors moderate the effect of local ones on the debt decisions of European SMEs and the second hypothesis is confirmed.

These findings answer an important and unresolved question: are SME debt policies more conditioned by LBD or NBD? This article demonstrates that the NBD reduces the effect of local banking institutions on SME debt choices. It seems that when national banking systems are more developed, the role of local sectors is lower. Thus, well-functioning credit sectors at the national scale reduce the relevance of local contexts. This result was observable also in Table 4 where in column 2 the standardized coefficients of the variable *NBD* are higher than the standardized coefficients of the variable *LBD*. Therefore, the national banking sectors drive European SME debt behavior more than the local ones. It seems that the role of the Central National Banks at the country level, together with the growth of globalization and the integration of financial markets, make the national banking sectors more influential than the local ones. However, this does not imply that the local context does not matter anymore, but it indicates that the debt policies of European SMEs are more favored by the development of the national banking sector. Consequently, local banking sectors still remain relevant, but to a lesser extent with respect to the national ones. Thus, answering the question that arises in the title of the present article, it is possible to conclude that national banking sectors win over local ones.

### Further and robustness tests

#### Further tests

As a first further test, following previous studies on corporate debt policies that draw attention to debt-maturity structure (Fan et al., 2012) also for SMEs (Hernandez-Canovas & Koeter-Kant, 2008; La Rocca et al., 2010), we distinguish between long- and short-term bank debt. Table 5 reports the corresponding output.

**Table 5:** Further test: results distinguishing between long and short term bank debt

Estimation technique:	(1) Panel FE	(2) Panel FE	(3) Panel FE	(4) Panel FE
Dependent variable	Short Debt	Short Debt	Long Debt	Long Debt
LBD	0.010	− 0.013	0.031***	0.030***
	(0.004)	(0.004)	(0.003)	(0.003)
NBD		0.035		0.002***
		(0.039)		(0.003)
Control variables	Yes	Yes	Yes	Yes
Adj. R2	0.176	0.179	0.210	0.215
Observations	285.974	285.974	285.974	285.974

Notes: Industry and year fixed effects are the controls. The superscripts denote significance as follows: *p* < 0.10, **p < 0.05, ***p < 0.01. Regressions report heteroscedastic robust standard errors in brackets. Full Table is available upon request to the authors

Table 5 shows a very interesting result. Both LBD and NBD seem to have no relevance on short-term bank debt ratios, while they significantly and positively impact on the use of long-term bank debt by SMEs. This is relevant, but not surprising. Indeed, as evidenced by the articles of Diamond (1991) and Barclay and Smith (1995), which represent two fundamental contributions in the financial literature, banks react to the underdevelopment of the financial sectors by reducing the maturity of their loans. Shorter loans allow banks to frequently monitor SMEs and interrupt the bank lending relationship if the firm becomes poor creditor. A longer loan maturity could cause greater losses in the event of insolvency. Therefore, where the banking systems (both local and national) are more developed and asymmetric information is limited, banks tend to increase debt maturity. Consequently, the development of local and national banking sectors is a matter of importance only for long-term bank debt choices.

As an additional further test, we examined whether the moderating effect of NBD changes before (the period 2004–2007) and during the Global Financial Crisis (the period 2008–2010). We observe that the moderating effect of the national banking system on SMEs’ use of debt highlighted in our second hypothesis does not significantly change before and during the crisis. Results are reported in Table 6.

**Table 6:** Further test: results before and after the global financial crisis

Estimation technique:	(1) Panel FE	(2) Panel FE	(3) Panel FE	(4) Panel FE
Dependent Variable	Debt Before 2008	Debt Before 2008	Debt After 2008	Debt After 2008
LBD	0.063***	0.002***	0.103***	0.047***
	(0.002)	(0.002)	(0.002)	(0.003)
NBD		0.148***		0.119***
		(0.002)		(0.002)
Control variables	Yes	Yes	Yes	Yes
Adj. R2	0.233	0.241	0.210	0.214
Observations	154.147	154.147	131.827	131.827

Industry and year fixed effects are the controls. The superscripts denote significance as follows: *p < 0.10, **p < 0.05, ***p < 0.01. Regressions report heteroscedastic robust standard errors in brackets.

Full Table is available upon request to the authors

As an additional further test, we collected data^17^ to investigate whether our results are confirmed using a database which considers a more recent period. Thus, we obtained data for the countries Italy and Spain^18^ for the period 2010–2016 and we re-run our main model. Table 7 shows the corresponding results that interestingly support the findings of our main model even in a more recent context.

**Table 7:** Further test: results concerning the effect of LBD on SME debt policies and the moderating effect of national on LBD in Italy and Spain for the period 2010–2016

Estimation technique:	(1) Panel FE	(2) Panel FE	(3) Panel FE
Dependent variable	Debt	Debt	Debt
LBD	0.100***	0.070***	0.175***
	(0.002)	(0.002)	(0.005)
NBD		0.000***	0.001***
		(0.000)	(0.000)
LBD × NBD			− 0.001***
(interaction)			(0.000)
Cash holdings	0.000***	0.000***	0.000***
	(0.000)	(0.000)	(0.000)
Trade credit	− 0.063***	− 0.062***	− 0.048***
	(0.002)	(0.002)	(0.003)
Interests	0.010***	0.010***	0.012***
	(0.004)	(0.004)	(0.004)
ROA	− 0.000**	− 0.000**	− 0.092***
	(0.000)	(0.000)	(0.001)
Size	0.031***	0.031***	0.037***
	(0.000)	(0.000)	(0.000)
Tangibility	0.144***	0.144***	0.140***
	(0.002)	(0.002)	(0.003)
Age	− 0.042***	− 0.042***	− 0.041***
	(0.001)	(0.001)	(0.001)
Diff. tax shield	− 0.003***	− 0.003***	− 0.002***
	(0.000)	(0.000)	(0.000)
	0.100***	0.070***	0.175***
Firm growth	(0.002)	(0.002)	(0.005)
		0.000***	0.001***
GDP growth		(0.000)	(0.000)
Enforcement	0.052	0.088*	0.098***
	(0.021)	(0.033)	(0.028)
Adj. R2	0.097	0.097	0.097
Observations	3.486.272	3.486.272	3.486.272

Industry and year fixed effects are the controls. The superscripts denote significance as follows: *p < 0.10, **p < 0.05, ***p < 0.01. Regressions report heteroscedastic robust standard errors in brackets

#### Robustness test: OLS two dimensions cluster technique

As a first robustness test,^19^ we run OLS two dimensions cluster technique, according to Petersen’s approach.^20^ This estimation procedure accounts for multiple dimensions at the same time (Cameron et al., 2008), as clustering allows to control for observations that are correlated under two dimensions (country and province). Hence, regressions correct the standard errors for the possible dependence of the residuals within clusters, as they consider that the variables measuring LBD and NBD vary at the provincial and at the national level, respectively. Results confirm the findings of the main model. This demonstrates once again that European SMEs benefit most from the development of national banking sectors.

#### Robustness test: endogeneity issue

We ran the Durbin-Wu-Hausman endogeneity test of endogenous regressors on the variables *LBD* and *NBD*. Although the test suggests the absence of an endogeneity issue, we also checked whether the results were still valid when controlling for this kind of bias by using the 2SLS technique. As previous studies based on Italy have settled endogeneity problems using regressions with instrumental variables (IV), we run 2SLS regressions on Italy using the same IV as in Guiso et al. (2004), La Rocca et al. (2010) and Fasano and Deloof (2021), who all measure local banking structures in 1936.^21^ The output of the 2SLS model, even if based on a single country, confirms our findings, as the regression results do not show substantial differences in terms of magnitude and statistical significance compared to our main model (results are available upon request to the authors).

In addition, we lagged all the independent variables in our regression analysis in order to roughly account in a different way for endogeneity concerns (Bellemare et al., 2017). The results provide robust estimates of the effects of the controls and confirm our findings.

#### Robustness test: placebo test

As a supplementary robustness test, we run the placebo test. Our sample has a very high number of observations that could affect the statistical significance of the findings (Athey & Imbens, 2017). To make sure that this number does not lead to false statistically significant results we applied a placebo test, in which 200 times we randomly assigned a branch density and a domestic credit provided by the financial sector by banks to each firm of the sample, and each time re-estimated the regression with the variables *LBD* and *NBD* reshuffled. We expect that in this setting LBD and NBD do not significantly influence SMEs’ use of debt. When we run the placebo test 200 times for each dependent variable, we find that the estimated coefficients of *LBD* and *NBD* are not statistically significant at the 10% level in more than 90% of the cases (results are available upon request to the authors). Hence, the results of placebo tests confirm the robustness of the findings, which are thus not influenced by chance.

#### Other robustness tests

In order to control whether our findings are driven by the presence of large financial centres, we drop from our dataset the biggest centers of each country (e.g., Frankfurt, Paris, Greater London, etc.). Regressions confirm the validity of the main model results. Moreover, we investigated our hypotheses by using a different proxy of LBD, i.e. *Loans_Deposits* calculated as the ratio of loans scaled to deposits collected by banks in the province. This measure has already been used as alternative measure of LBD by the extant literature (e.g. Deloof & La Rocca, 2015). Results confirm the findings observed in our main model.

## Conclusion and discussion

Beginning with the milestone contribution of Guiso et al. (2004), the financial literature studied the relevance of LBD for firm growth, with a particular attention on SMEs. While previous studies focus on single country settings of analysis, in a more innovative manner the present article investigates whether local banking sectors matter in a wide European context. The empirical results reveal that the development of local banking institutions significantly and positively influences the debt policies of European SMEs. In more detail, we find that the geographic proximity of bank branches has a key role in the financing of SMEs, as it reduces information asymmetries and facilitates credit provision. The findings, which are supported by a number of robustness tests, interestingly reveal that LBD plays an important role in SME debt policies in the larger European context.

Assuming that LBD is also relevant in a cross-country environment, this research addresses another important question still unresolved: do European SMEs benefit most from local or NBD? The findings reveal that, notwithstanding the relevance of the local contexts, as indicated by the extant literature, the national dimension of the banking system seems to better drive the debt policies of European SMEs. In particular, our moderation study highlights that the relative importance of LBD decreases with higher levels of NBD. Consequently, in those contexts where the national banking institutions are more developed, local credit markets have a lesser influence on SME debt policies. Hence, although local bank branches are still important, SMEs rely more on the development of the banking macro-system of a country.

Our paper is at the crossroads of two lines of literature that in parallel study local (within country) and national (between countries) banking development and their effects on corporate debt policies. At the same time, it advances these strands of research, as it investigates for the first time SME debt strategies considering both local and national banking institutions in a cross-country European setting. The results highlight that the national banking environment moderates the role of the provincial banking system, suggesting that future researchers in this field take into account the macro‐level contingencies in which SMEs are embedded. Thus, the key findings of this article concern the need to joint consider LBD and NBD as crucial determinants of debt decisions of European SMEs. Additionally, further research could explore to which tipping point the level of NBD does not influence the role of LBD. A limitation of this study is the period of analysis that does not consider recent years. Further studies could thus investigate the effect of LBD and NBD for firms during the pre and covid years.

Moreover, the work has also implications for policy makers and researchers. Given that national rather than LBD resulted in being more effective in influencing the debt choices of European SMEs, governments should primarily commit to improving the national banking institutions that drive the operations of local banking sectors. Regulations aimed at developing and making more effective national banking sectors are important to ensure SMEs a priority channel to external resources. According to our findings, the banking institutions of a country should act as a fellow traveller that accompanies SMEs towards value creation processes, overcoming obstacles and external frictions for SMEs. This is especially important during the current Covid-19 downturn, where European SMEs are in great need of financial resources to recover from the crisis. In this regard, we recommend that policy makers enhance the effectiveness of the banking systems, especially the national ones, in order to meet the credit need of small European businesses. Decision makers could improve European SME access to outside sources of financing by creating incentive policies and specialized credit patterns for SMEs. The presence of strong national banking institutions should be encouraged. The European Commission and the governments of the individual countries of the European Union should move in this direction, with the final aim to reduce the asymmetric information problems of SMEs and, more generally, all the financial constraints that impede corporate growth. Moreover, governments should upgrade the role of the banking institutions, making them not only credit providers, but also financial advisers that can provide support in understanding appropriate financial strategies. This approach could increase not only the quantity, but also the quality of external funds available for SMEs. In more detail, national banking institutions could establish a program aimed at providing professional financial advice for SMEs. Such a program needs to be applied at the local level in order to bridge the gap of financial skills that SMEs need. Indeed, this is particularly important, as the managers of SMEs are often in need of more financial skills (Van Auken, 2005) that could affect the quality of their financial decisions.

Our work suggests that the local banking sector is still important, despite the internalization and integration of financial markets, and it can significantly support SME businesses. Local bank branches are directly in charge of collecting and interpreting soft information from their relationship with SMEs in credit provision. Agarwal and Hauswald (2010, p. 2759) point out that “soft information in credit markets is primarily local”. Indeed, the local banking system is still the “front office” that meets SMEs requests. Thus, the effectiveness of LBD is essential to reduce the financial knowledge gap. In this context, well-developed banking institutions should not only guarantee the availability of liquidity in the presence of high profits and collaterals, but also continuously support SMEs from a financial point of view. Thus, the growth of the national banking industry should be balanced by the contemporary growth in efficiency of the provincial banking sectors. The banking headquarters and the central banks could direct local branches in conceding credits in order to spur LBD. In particular, they could better support local branches in integrating national banking policies. An example of how the national system drives the local is the use of credit scoring techniques that local branches are required to employ in their loan decision process. The empirical evidence shows that SMEs remain dependent at the level of LBD, recognizing that the big (national) context has a main role over the small (local) one in determining the debt decisions of small businesses. Thus, another implication of our article is that when NBD is low, policy makers should focus on developing the local banking system, which has a greater importance to SMEs in this context.

In conclusion, in the current context where much attention is paid to the financial resources available for SMEs in severe difficulty caused by the coronavirus crisis, it is of importance that European policy makers formulate policies that promote the development of national banking institutions. Indeed, “the state of development of the financial markets does indeed facilitate growth, and is not simply correlated with it” (Rajan & Zingales, 2001, p. 470). Therefore, banking development should be strongly encouraged as it could positively affect the growth of the millions of European SMEs and, in turn, of the entire European economy.

## Data Availability

The accounting data that support the findings of this study are available from third party: Bureau Van Dijk. Restrictions apply to the availability of these data, which were used under license for this study, and we not allowed to publicy deposit the data.

## Appendix

See Appendix Table 8, 9, 10, 11, 12, 13.

**Table 8:** Descriptive Statistics of debt for high and low levels of LBD based on median

	High LBD						
	Mean	Sd	Min	p25	Median	p75	Max
Debt	0.216	0.204	0.000	0.025	0.168	0.359	0.847
	Low LBD						
	Mean	Sd	Min	p25	Median	p75	Max
Debt	0.188	0.188	0.000	0.017	0.133	0.317	0.847

**Table 9:** Mean and median of debt for high and low levels of NBD based on median

	High *NBD*						
	Mean	Sd	Min	p25	Median	p75	Max
Debt	0.204	0.203	0.000	0.020	0.146	0.337	0.847
	Low *NBD*						
	Mean	Sd	Min	p25	Median	p75	Max
Debt	0.198	0.192	0.000	0.020	0.149	0.335	0.847

**Table 10:** Number of firms by country

Sector	Number of obs	Per. cum
Finland	5,777	2,02%
France	48,314	18,91%
Germany	8,765	21,98%
Italy	133,613	68,70%
Spain	68,912	92,80%
United Kingdom	20,593	100,00%
Total	285,974	

**Table 11:** Number of firms by sector

Sector	Number of obs	Perc. cum
Agriculture, forestry and fishing	64,371	23,31%
Manufacturing, mining and quarrying and other industry	135,497	68,26%
Construction	24,016	77,04%
Wholesale and retail trade, transportation and storage, accommodation and food service activities	24,109	86,23%
Information and communication	24,614	94,83%
Financial and insurance activities	13,367	100,00%
Total	285,974	

**Table 12:** Descriptive statistics by country

	Mean	Sd	Min	p25	Median	p75	Max
Finland							
Debt	0.227	0.203	0.000	0.036	0.191	0.363	0.847
LBD	0.312	0.139	0.172	0.212	0.283	0.334	1.142
NBD	0.772	0.079	0.637	0.692	0.763	0.862	0.879
Cash Holdings	0.092	0.116	0.000	0.011	0.048	0.131	0.844
Trade Credit	0.070	0.131	− 0.603	− 0.003	0.059	0.130	0.969
ROA	0.091	0.135	− 0.317	0.020	0.084	0.166	0.434
Size	8.136	0.847	1.946	7.521	8.078	8.693	10.667
Tangibility	0.336	0.224	0.000	0.143	0.323	0.506	0.988
Age	2.763	0.816	0.000	2.398	2.833	3.258	4.718
Different Tax Shield	0.058	0.030	0.002	0.035	0.055	0.083	0.104
Firm Growth	− 0.081	0.317	− 5.834	− 0.179	− 0.053	0.055	0.767
GDP Growth	0.162	0.010	0.138	0.155	0.159	0.166	0.180
Enforcement	0.028	0.006	0.023	0.023	0.023	0.038	0.038

France							
Debt	0.100	0.129	0.000	0.007	0.052	0.144	0.847
LBD	0.582	0.156	0.250	0.462	0.571	0.682	2.253
NBD	0.869	0.069	0.767	0.801	0.888	0.925	0.958
Cash Holdings	0.140	0.159	0.000	0.020	0.079	0.212	0.979
Trade Credit	0.073	0.193	− 2.689	− 0.034	0.081	0.190	0.953
ROA	0.071	0.123	− 0.317	0.014	0.062	0.130	0.434
Size	8.341	0.913	5.070	7.660	8.284	8.983	10.666
Tangibility	0.175	0.162	0.000	0.056	0.127	0.243	0.978
Age	3.013	0.840	0.000	2.485	3.135	3.611	5.347
Different Tax Shield	0.041	0.028	0.002	0.019	0.034	0.057	0.104
Firm Growth	− 0.057	0.269	− 13.359	− 0.127	− 0.037	0.046	0.902
GDP Growth	0.254	0.010	0.225	0.246	0.252	0.259	0.271
Enforcement	0.039	0.000	0.039	0.039	0.039	0.039	0.039

Germany							
Debt	0.239	0.219	0.000	0.029	0.199	0.389	0.847
LBD	0.574	0.129	0.255	0.444	0.580	0.716	1.116
NBD	0.965	0.048	0.885	0.970	0.971	0.988	1.064
Cash Holdings	0.100	0.136	0.000	0.006	0.044	0.139	0.923
Trade Credit	0.066	0.145	− 1.102	− 0.008	0.052	0.131	0.822
ROA	0.085	0.126	− 0.317	0.027	0.074	0.141	0.434
Size	9.080	0.854	5.548	8.561	9.131	9.726	10.668
Tangibility	0.328	0.243	0.000	0.110	0.292	0.512	0.994
Age	2.904	0.850	0.000	2.398	2.890	3.466	4.745
Different Tax Shield	0.052	0.031	0.002	0.026	0.050	0.076	0.104
Firm Growth	− 0.054	0.318	− 11.953	− 0.143	− 0.030	0.069	0.923
GDP Growth	0.190	0.010	0.136	0.183	0.192	0.199	0.201
Enforcement	0.039	0.000	0.039	0.039	0.039	0.039	0.040

Italy							
Debt	0.226	0.190	0.000	0.035	0.208	0.374	0.847
LBD	0.655	0.154	0.212	0.571	0.665	0.763	1.060
NBD	0.809	0.080	0.674	0.755	0.815	0.874	0.929
Cash Holdings	0.071	0.103	0.000	0.004	0.027	0.094	0.932
Trade Credit	0.092	0.181	− 1.802	− 0.005	0.084	0.195	0.954
ROA	0.053	0.081	− 0.317	0.018	0.043	0.079	0.434
Size	8.881	0.824	5.161	8.279	8.897	9.492	10.669
Tangibility	0.225	0.188	0.000	0.070	0.180	0.336	0.993
Age	2.943	0.733	0.000	2.565	3.091	3.434	4.997
Different Tax Shield	0.038	0.025	0.002	0.019	0.032	0.050	0.104
Firm Growth	− 0.055	0.301	− 17.054	− 0.150	− 0.034	0.077	0.947
GDP Growth	0.238	0.010	0.211	0.231	0.238	0.243	0.256
Enforcement	0.125	0.007	0.121	0.121	0.121	0.121	0.139

Spain							
Debt	0.201	0.205	0.000	0.016	0.139	0.337	0.847
LBD	1.021	0.178	0.555	0.895	1.006	1.094	1.669
NBD	1.550	0.207	1.160	1.360	1.679	1.720	1.740
Cash Holdings	0.111	0.141	0.000	0.016	0.055	0.149	0.989
Trade Credit	0.254	0.234	− 2.451	0.084	0.221	0.399	0.999
ROA	0.060	0.099	− 0.317	0.018	0.048	0.097	0.434
Size	8.526	0.953	3.369	7.809	8.476	9.226	10.668
Tangibility	0.237	0.218	0.000	0.063	0.173	0.351	0.996
Age	2.801	0.701	0.000	2.398	2.890	3.258	4.820
Different Tax Shield	0.038	0.029	0.002	0.014	0.030	0.055	0.104
Firm Growth	− 0.057	0.372	− 14.996	− 0.149	− 0.025	0.098	0.935
GDP Growth	0.245	0.012	0.213	0.236	0.242	0.257	0.266
Enforcement	0.052	0.000	0.052	0.052	0.052	0.052	0.052

United Kingdom							
Debt	0.269	0.240	0.000	0.063	0.205	0.422	0.847
LBD	0.853	0.401	0.301	0.511	0.826	1.057	1.884
NBD	1.686	0.209	1.369	1.449	1.694	1.906	1.921
Cash Holdings	0.115	0.145	0.000	0.011	0.059	0.167	0.991
Trade Credit	0.106	0.150	− 1.140	0.017	0.096	0.182	0.916
ROA	0.067	0.132	− 0.317	0.006	0.062	0.133	0.434
Size	8.914	0.782	5.952	8.394	8.920	9.478	10.668
Tangibility	0.241	0.202	0.000	0.075	0.190	0.363	0.984
Age	2.964	0.837	0.000	2.398	3.045	3.555	4.883
Different Tax Shield	0.041	0.028	0.002	0.019	0.035	0.057	0.104
Firm Growth	− 0.055	0.343	− 9.975	− 0.152	− 0.023	0.103	0.908
GDP Growth	0.245	0.011	0.218	0.238	0.241	0.249	0.266
Enforcement	0.040	0.000	0.040	0.040	0.040	0.040	0.040

**Table 13:** Descriptive statistics by industry

Agriculture, forestry and fishing	Mean	Sd	min	p25	Median	p75	max
Debt	0.224	0.195	0.000	0.042	0.191	0.367	0.847
LBD	0.726	0.266	0.172	0.558	0.699	0.859	2.253
NBD	1.014	0.351	0.637	0.801	0.874	1.160	1.921
Cash Holdings	0.082	0.118	0.000	0.006	0.034	0.109	0.991
Trade Credit	0.109	0.186	− 2.431	0.000	0.098	0.212	0.956
ROA	0.048	0.086	− 0.317	0.013	0.040	0.079	0.434
Size	8.720	0.873	5.146	8.083	8.733	9.366	10.668
Tangibility	0.260	0.191	0.000	0.103	0.222	0.382	0.983
Age	3.057	0.753	0.000	2.708	3.178	3.555	5.347
Different Tax Shield	0.042	0.027	0.002	0.021	0.036	0.057	0.104
Firm Growth	− 0.039	0.251	− 13.359	− 0.115	− 0.022	0.067	0.927
GDP Growth	0.239	0.018	0.136	0.232	0.239	0.251	0.271
Enforcement	0.085	0.041	0.023	0.040	0.121	0.121	0.139

Manufacturing, mining and quarrying and other industry							
Debt	0.192	0.187	0.000	0.021	0.141	0.324	0.847
LBD	0.714	0.243	0.172	0.560	0.699	0.837	2.253
NBD	1.018	0.355	0.637	0.801	0.874	1.054	1.921
Cash Holdings	0.092	0.121	0.000	0.008	0.042	0.130	0.979
Trade Credit	0.126	0.184	− 1.877	0.017	0.116	0.223	0.991
ROA	0.063	0.097	− 0.317	0.020	0.052	0.103	0.434
Size	8.756	0.849	5.505	8.121	8.757	9.387	10.669
Tangibility	0.214	0.169	0.000	0.076	0.174	0.316	0.994
Age	2.984	0.737	0.000	2.639	3.091	3.466	5.273
Different Tax Shield	0.040	0.025	0.002	0.020	0.034	0.053	0.104
Firm Growth	− 0.057	0.295	− 13.285	− 0.160	− 0.041	0.079	0.947
GDP Growth	0.240	0.019	0.136	0.234	0.241	0.253	0.271
Enforcement	0.085	0.042	0.023	0.040	0.121	0.121	0.139

Construction							
Debt	0.218	0.197	0.000	0.036	0.178	0.357	0.847
LBD	0.716	0.251	0.172	0.569	0.703	0.845	2.253
NBD	1.046	0.378	0.637	0.815	0.874	1.360	1.921
Cash Holdings	0.089	0.119	0.000	0.008	0.041	0.123	0.887
Trade Credit	0.120	0.187	− 1.030	0.008	0.103	0.219	0.975
ROA	0.060	0.098	− 0.317	0.018	0.049	0.098	0.434
Size	8.799	0.889	3.369	8.150	8.809	9.464	10.668
Tangibility	0.241	0.204	0.000	0.075	0.183	0.358	0.972
Age	2.870	0.760	0.000	2.485	2.996	3.367	5.323
Different Tax Shield	0.040	0.025	0.002	0.020	0.034	0.054	0.104
Firm Growth	-0.058	0.333	− 15.368	− 0.142	− 0.033	0.073	0.931
GDP Growth	0.236	0.020	0.144	0.230	0.239	0.247	0.271
Enforcement	0.088	0.041	0.023	0.040	0.121	0.121	0.139

Wholesale and retail trade, transportation and storage, accommodation and food service activities							
Debt	0.214	0.205	0.000	0.025	0.160	0.359	0.847
LBD	0.836	0.256	0.212	0.665	0.853	1.006	1.669
NBD	1.249	0.386	0.674	0.835	1.360	1.679	1.740
Cash Holdings	0.096	0.130	0.000	0.010	0.044	0.129	0.989
Trade Credit	0.184	0.276	− 2.451	0.000	0.132	0.355	0.989
ROA	0.061	0.080	− 0.317	0.024	0.047	0.085	0.434
Size	8.678	0.997	4.606	7.917	8.663	9.448	10.669
Tangibility	0.130	0.147	0.000	0.028	0.079	0.179	0.994
Age	2.686	0.735	0.000	2.197	2.773	3.219	4.700
Different Tax Shield	0.019	0.020	0.002	0.006	0.013	0.025	0.104
Firm Growth	− 0.087	0.504	− 14.967	− 0.237	− 0.018	0.177	0.935
GDP Growth	0.238	0.013	0.171	0.231	0.238	0.243	0.266
Enforcement	0.079	0.036	0.039	0.052	0.052	0.121	0.139

Information and communication							
Debt	0.202	0.229	0.000	0.002	0.113	0.339	0.847
LBD	0.783	0.286	0.172	0.580	0.791	0.998	2.253
NBD	1.204	0.397	0.637	0.842	0.958	1.679	1.921
Cash Holdings	0.138	0.177	0.000	0.014	0.062	0.193	0.969
Trade Credit	0.059	0.223	− 2.689	− 0.046	0.019	0.142	0.985
ROA	0.069	0.140	− 0.317	0.007	0.050	0.125	0.434
Size	8.391	1.069	1.946	7.595	8.397	9.184	10.667
Tangibility	0.354	0.299	0.000	0.075	0.281	0.598	0.996
Age	2.747	0.808	0.000	2.197	2.833	3.296	5.257
Different Tax Shield	0.051	0.033	0.002	0.023	0.046	0.077	0.104
Firm Growth	− 0.043	0.265	− 12.303	− 0.095	− 0.023	0.048	0.933
GDP Growth	0.246	0.020	0.136	0.238	0.248	0.257	0.271
Enforcement	0.061	0.033	0.023	0.039	0.052	0.052	0.139

Financial and insurance activities							
Debt	0.144	0.192	0.000	0.000	0.050	0.239	0.847
LBD	0.744	0.291	0.172	0.531	0.698	0.913	1.884
NBD	1.176	0.412	0.637	0.835	0.950	1.679	1.921
Cash Holdings	0.146	0.167	0.000	0.019	0.083	0.217	0.926
Trade Credit	0.254	0.255	− 1.393	0.074	0.234	0.417	0.999
ROA	0.076	0.141	− 0.317	0.017	0.065	0.142	0.434
Size	8.459	0.900	5.342	7.790	8.421	9.115	10.666
Tangibility	0.108	0.149	0.000	0.018	0.046	0.132	0.933
Age	2.475	0.674	0.000	2.079	2.485	2.996	4.718
Different Tax Shield	0.040	0.033	0.002	0.013	0.029	0.060	0.104
Firm Growth	− 0.100	0.402	− 17.054	− 0.186	-0.050	0.056	0.883
GDP Growth	0.246	0.021	0.136	0.238	0.251	0.259	0.271
Enforcement	0.070	0.038	0.023	0.039	0.052	0.121	0.139

## Footnotes

1 The financial system is intended as the set of instruments, institutions and mechanisms that ensure the transfer of financial resources from surplus to deficit subjects (financial resources allocation function).

2 The term “banking development” indicates the development of those parts of the financial system composed of banking institutions. LBD refers to the banking development at the provincial or regional level.

3 According to Petersen (2004, p. 1), “hard information is quantitative, easy to store and transmit in impersonal ways, and its content is independent of the collection process,” whereas soft information “is difficult to completely summarize in a numeric score.”.

4 European Central Bank, Economic Bulletin, Issue 4/2020, article “Access to finance for small and medium-sized enterprises since the financial crisis: evidence from survey data”, prepared by Katarzyna Bańkowska, Annalisa Ferrando and Juan Angel Garcia. Available at https://www.ecb.europa.eu/pub/economic-bulletin/articles/2020/html/ecb.ebart202004_02~80dcc6a564.en.html#toc1

5 https://ec.europa.eu/growth/smes/sme-definition_en

6 A deeper description of the banking systems of the countries analyzed is reported in Paragraph 3.1.

7 World Development Indicators is the World Bank collection of development indicators, compiled from officially recognized international sources.

8 (https://data.worldbank.org/indicator/FS.AST.DOMS.GD.ZS).

9 The sample consists of these six nations due to the difficulty in accessing LBD information about other EU countries. Moreover, more recent data about LBD in UK was not anymore available.

10 EUR-LEX: 2003/361/EC: Commission Recommendation of 6 May 2003 concerning the definition of micro, small and medium-sized enterprises, *Official Journal of the European Union* L124/36, 20.5.2003, pp. 36–41.

11 Statistics Finland and Courts of Appeal (Finland), INSEE (France), Federal Statistical Office Germany (Germany), ISTAT (Italy), INE and Consejo General del Poder Judicial (Spain), Office for National Statistics (United Kingdom).

12 Number of observations per country and sector is reported in Tables 10 and 11 in the appendix.

13 Suomen Pankki (Finland), Banque de France (France), Deutsche Bundesbank (Germany), Banca d’Italia (Italy), Banco de España (Spain), Bank of England (United Kingdom).

14 European Central Bank, *Economic Bulletin*, Issue 4/2020, article “Access to finance for small and medium-sized enterprises since the financial crisis: evidence from survey data”, prepared by Katarzyna Bańkowska, Annalisa Ferrando and Juan Angel Garcia. Available at https://www.ecb.europa.eu/pub/economic-bulletin/articles/2020/html/ecb.ebart202004_02~80dcc6a564.en.html#toc1

15 We also observed the differences based on the 25th and 75th percentiles, obtaining the same results.

16 Figure 2 is based on the Jeremy Dawson graphs (www.jeremydawson.co.uk/slopes.htm).

17 The process of data collection is the same as indicated in Paragraph 3.1.

18 It was not possible to collect data for other countries because information required was not available.

19 Regression results of robustness tests are available upon request to the authors.

20 We used the Mitchell Petersen’s Stata routine cluster standard errors by two dimensions (available at https://www.kellogg.northwestern.edu/faculty/petersen/htm/papers/se/se_programming.htm).

21 These structures were established by factors unrelated to local economic development. The legislation introduced in Italy in 1936 limited the growth of the national banking sector, for which provincial differences present in 1936 still exist today. The instruments consist of the amount of bank branches in 1936, the number of banks, the number of popular bank branches, and the 1936 branch density in the SMEs’ province. Popular banks are larger cooperative banks. It is generally hard to get valid instrumental variables since the factors that are probably most closely linked with the endogenous variable are other characteristics that are, potentially, included in the regressions as control variables. Therefore, we decided to run the 2SLS as a robustness only on the Italian context using the same IVs of previous studies.
